# Primary Antimicrobial Susceptibility Changes in Children with *Helicobacter pylori* Infection over 13 Years in Northern Italy

**DOI:** 10.1155/2015/717349

**Published:** 2015-05-06

**Authors:** Marco Manfredi, Pierpacifico Gismondi, Valentina Maffini, Barbara Bizzarri, Fabiola Fornaroli, Carmen Madia, Antonino Salerno, A. Marta Cangelosi, Gian Luigi de'Angelis

**Affiliations:** ^1^Department of Pediatrics, “Pietro Barilla” Children's Hospital, Azienda Ospedaliero-Universitaria di Parma, University Hospital, Via Gramsci 14, 43126 Parma, Italy; ^2^Gastroenterology and Endoscopy Unit, Azienda Ospedaliero-Universitaria di Parma, University Hospital, Via Gramsci 14, 43126 Parma, Italy

## Abstract

The eradication therapy of *Helicobacter pylori* (*H. pylori*) infection is still a challenge for gastroenterologists. One of the main causes of failure in *H. pylori* eradication is the antibiotic resistance mainly to clarithromycin. Culture from biopsies is maybe the most used method among the antimicrobial susceptibility techniques. In this study, we compared the antimicrobial susceptibility changes in children with *H. pylori* infection over 13 years and we confirmed that clarithromycin resistance has been increased (16% versus 26%) though with no statistically signficant value. Therefore, clarithromycin should not be used in empiric treatment of *H. pylori* eradication therapy in children, but its use should be limited only to children with known antimicrobial susceptibility. On the other hand, metronidazole resistance has decreased over this time period in statistically significant manner (56% versus 33%, *p* = 0.014). Furthermore, ampicillin resistance has been confirmed to be very rare (3% versus 0%) in children with *H. pylori* infection. In conclusion, in *H. pylori* infection, if we do not know the antibiotic susceptibility of patients, we should recommend an eradication therapy based on the local distribution of antibiotic resistance rates trying to limit the therapeutic failures.

## 1. Introduction

*Helicobacter pylori* (*H. pylori*) infection is one of the most common infections in humans. Although many efforts have been made in trying eradication, several difficulties remain to be overcome.

For many years the first-line therapy recommended by the international guidelines in eradication of* H. pylori* infection has been the triple therapy (PPI + amoxicillin + clarithromycin or metronidazole).

During the last years, the widespread use/abuse of antibiotics, particularly for respiratory tract infections, led to the emergence of increasing resistance of* H. pylori* infection to common antibiotics, mainly to clarithromycin that is almost doubled over the past 10 years, from 9.8% to 17.5% [1]. In fact, currently, the triple therapy usually achieves an eradication rate less than 80%, the minimum eradication rate universally accepted. For these reasons, Maastricht IV Conference recommended abandoning clarithromycin in empirical eradication therapy or testing susceptibility for this antibiotic before using it, if its local resistance prevalence is higher than 15–20%. In this case, the recommended therapy in adults is the quadruple therapy with or without bismuth compounds or alternatively the sequential therapy [1]. Furthermore, several studies showed that sequential therapy can overcome the resistance to clarithromycin both in adults and in children [2, 3].

Actually, before starting an eradication treatment, we should also consider the resistance to quinolones because it is increasing worldwide over the last years [4].

Besides, also the resistance to metronidazole is often high, but it can be overcome by increasing the duration of treatment or by prescription of bismuth-containing quadruple therapy including metronidazole [1].

Culture is the most common and specific invasive diagnostic method to detect the antimicrobial susceptibility of* H. pylori*, although its sensitivity can be largely variable (60–90%) [5], mainly due to several methodological factors such as biopsy site, transport medium, time from sampling to processing, culture medium, and incubation conditions. Usually the culture of a single antral biopsy allows the detection of* H. pylori* in more than 90% of the cases, but for optimal results and in accordance with the updated Sydney system [6] at least one biopsy from antrum and two biopsies from corpus should be taken for culture to ensure a correct diagnosis. The type of transport medium can affect the recovery rate of* H. pylori*, but the transportation time seems to be the more critical. Using Portagerm pylori cultured rates range from 77 to 94% [7].

In children ESPGHAN/NASPGHAN guidelines recommend antibiotic susceptibility testing for clarithromycin before starting clarithromycin-based triple therapy in areas/populations with a known high resistance rate (>20%) [8]. In Italy the clarithromycin resistance is around 25% both in adults [9] and in children [10].

The most common antibiotics used in* H. pylori *eradication treatment in children are amoxicillin, clarithromycin, and metronidazole (or imidazole) [8]. The use of quinolones in children is limited because juvenile animals developed arthropathy in previous experiments on fluoroquinolone use, although these have not been clearly demonstrated in humans yet. However, their use in pediatric population has increased in many circumstances; quinolones may be particularly helpful in treating multidrug-resistant infections that have not responded to standard antibiotic therapy in immunocompromised patients [11].

Therefore, looking for the best eradication therapy, if the antimicrobial susceptibility is not available, we should ask patients which and how many antibiotics they used before, because previous antibiotics could negatively influence the efficacy of* H. pylori* treatment [12].

## 2. Aim

We wanted to evaluate the variations in primary antibiotic susceptibility over the last 13 years in children with* H. pylori* infection in Parma, northern Italy, compared with our previous results obtained in 2001 [13].

## 3. Methods

### 3.1. Patients

From January 2011 to December 2012 we diagnosed by endoscopy and histological examination 66 naïve children with* H. pylori* infection aged between 39 and 192 months (16 years old) as reported in Table 1. Exclusion criteria were previous* H. pylori* treatments and therapies with either proton pump inhibitors or antibiotics in the 4 weeks before gastroscopy.

Clinical and endoscopical characteristics of patients are reported in Table 2.

Most of patients were subjected to EGDS because of recurrent abdominal pain (40.9%) and the most representative endoscopic feature was nodular gastritis.

### 3.2. Endoscopy

Endoscopic biopsy specimens were taken in all subjects for histology following Sydney Criteria (two from the gastric antrum and two from the gastric corpus-fundus) and microbiological culture (one from the antrum).

### 3.3. Histology/Culture

The biopsy specimens for histology were fixed in formalin, embedded in paraffin, sectioned, and stained with haematoxylin-eosin. The biopsy specimens for the bacterial culture were immediately placed in an appropriate transport medium (Portagerm-Pylori, bioMérieux, France) and then homogenised and cultured on both selective (Pylori agar, bioMérieux) and nonselective (10% horse blood agar, Kima, Italy) media. After seven days of incubation at 37°C under microaerophilic conditions, typical oxidase and catalase positive colonies were identified by API Campy strips (bioMérieux) and subsequently tested for antibiotic sensitivity (*E* test) [14]. The following antibiotics were tested: ampicillin, tetracycline, metronidazole, and clarithromycin (AB Biodisk, Sweden). The minimum inhibitory concentration (MIC) interpretative values used to define resistance (*R*) to each antibiotic are reported in Table 3.

Histologically all patients had chronic nonatrophic gastritis.

## 4. Results

Culture developed in 46/66 patients (70%) with a reduction of 14% compared to those performed 13 years before with no statistically significant value.

Throughout the last 13 years, we obtained a significant reduction in metronidazole resistance (56% versus 33%), while the clarithromycin resistance evidently increased though with no statistically significant value (16% versus 26%) (Table 3).

During these years, the resistance to ampicillin has been confirmed to be very low or absent as well as that to tetracyclines; in the same way the combined resistance to metronidazole and clarithromycin together has not been changed, staying very low.

## 5. Discussion

The eradication of* H. pylori* infection represents an enormous challenge in gastroenterology. Considering that this organism lives in an environment not easily accessible to many drugs, the increasing antibiotic resistance is a burden that we must fight every day.

To know the local prevalence of antibiotic resistance is important also for choosing the better therapy mainly if the antimicrobial susceptibility does not develop from the culture [9]; therefore we must use an empirical eradication treatment.

Culture of* H. pylori* can be difficult to obtain and its sensitivity has a wide range of variability despite its high cost. Our study shows a decline of culture development of* H. pylori *over the last 13 years: from 84% in 1998/99 to 70% in 2011/12 although there is not a statistically significant difference.

Culture can turn out to be negative even in the presence of positive urease test despite three biopsy samples took, as shown by Porowska et al [15]. These authors obtained a culture rate like ours: only 62% of patients examined had a positive culture. Furthermore they found a very high resistance rate to metronidazole, levofloxacin, clarithromycin, amoxicillin, and tetracycline (97%, 58%, 55%, 29%, and 23%, resp.) in adult patients.

On the contrary, other Italian researchers obtained very high culture development rate of 94% in adult patients using two antral biopsies for culture examination [16].

Chronic PPI intake is considered to be the main cause of culture failure and when considering options for susceptibility testing, biopsy specimens should also be taken from the gastric body [17].

But altogether, perhaps the main reason affecting the culture development remains the intrinsic difficulty of this technique [7].

Treatment failures due to drug-resistant* H. pylori* strains have become an increasing problem and this has prompted clinicians to prescribe alternative antimicrobial regimens, but unfortunately we do not have a wide range of antibiotics, mainly in children. The last ESPGHAN/NASPGHAN guidelines recommend not using clarithromycin in empirical triple therapy in areas with the clarithromycin resistance rate higher than 20%. Alternatively bismuth-containing quadruple therapy (bismuth + amoxicillin + clarithromycin + metronidazole) or sequential therapy is good first-line empirical choice in children [3, 8, 18]. In the last years, several studies reported high clarithromycin and metronidazole resistance rate in pediatric population compared to those in adults [19], which is correlated with the frequency of prior antibiotic prescription mainly due to respiratory tract infections treatment.

Thus, we should pay more attention to treatment by knowing the local antimicrobial susceptibility prevalence for increasing the successful eradication rate. Therefore clarithromycin-based triple therapy should be performed only if susceptibility testing in the individual patient revealed a clarithromycin-susceptible strain or if local clarithromycin resistance rate is known to be low (<10%) as recommended by last Maastricht [1] and NASPGHAN/ESPGHAN [8] guidelines.

Comparing our study with the previous one published in 2001, we obtained both a clear decrease in culture development rate and some changes in antibiotic resistance rate over the last 13 years. Metronidazole resistance decreased (from 56% to 33%) with statistically significant value, and that of clarithromycin increased (16% to 26%) (no statistically significant difference). However the frequency of both clarithromycin and metronidazole resistant strains together did not statistically change as also reported in a study from South Korea and Austria. [20, 21].

Also Kalach and coworkers showed a decrease in metronidazole resistant strains but they did not get changes in clarithromycin resistance in French children over 11 years [22] while, on the other hand, Oleastro et al. revealed no changes in clarithromycin nor in metronidazole resistance over a 10-year period in Portuguese children [23].

In our study, we confirmed that the resistance rate of* H. pylori* to amoxicillin is very rare around the world [24] as well as in recent studies from France, Austria, Spain, Portugal, and Japan [25]. In fact all eradication therapies of* H. pylori* infection include penicillin. However studies from South Korea surprisingly showed an increase in amoxicillin resistance rate over 9 years [20, 26].

It is well established that one of the main causes of* H. pylori* treatment failure is primary resistance towards antibiotics [27] which have been increased all over the world [28]; therefore the eradication rate following the standard treatments is clearly lower whether clarithromycin or metronidazole resistance is present [29].

In this study we confirmed the high resistance rate to clarithromycin in Italy [8]; therefore maybe as recommended by ESPGHAN/NASPGHAN guidelines in these cases we should use the bismuth-containing quadruple therapy as first-line empirical treatment. Also sequential therapy could be effective because this approach has been shown to overcome the clarithromycin resistance [2].

In our centre, we took a single biopsy specimen from the antrum for the* H. pylori* culture, and maybe this could be adjusted by adding another one/two samples also from the gastric body [17] trying to improve the culture successful rate.

But considering the difficulty of* H. pylori* culture development, this technique could now be substituted by molecular methods (as FISH or PCR from biopsies) which do not require strict conditions of biopsy specimens transport and can be used to detect the point mutation associated with the clarithromycin resistance, the main antibiotic responsible for decreasing of eradication rate [7].

In conclusion, as stated by many authors, we would like to stress the concept that, before recommending* H. pylori* eradication therapy, we should know either the antibiotic susceptibility of patient or the local distribution of antibiotic resistance rates to have higher successful probabilities of eradication.

## Figures and Tables

**Table 1:** Demographic characteristics of patients.

Sex	Range (months)	Mean age
Male: 37 pts and female: 29 pts	39–192 months	9 years and 8 months

**Table 2:** Clinical and endoscopical characteristics of patients.

Symptoms		Endoscopical aspects	
Recurrent abdominal pain	27 pts (40.9%)	Nodular Gastritis	52 pts (78.7%)
Epigastric pain	19 pts (28.8%)	Duodenal Ulcer	3 pts (4.5%)
Vomiting	9 pts (13.6%)	Duodenal Erosion	2 pts (3%)
Gastric pyrosis	6 pts (9.1%)	Nodular Duodenitis	9 pts (13.6%)
Anemia	5 pts (7.6%)		

**Table 3:** Differences in antibiotic resistance.

Antibiotic	MIC interpretative values	Resistance rate	Resistance rate	*p* value
	(*μ*g/mL)	(years 1998-99)	(years 2011-12)	
Metronidazole	>16	35 (56%)	15 (33%)	0.014
Clarithromycin	>4	10 (16%)	12 (26%)	0.142
Ampicillin	>4	2 (3%)	0	ns
Tetracyclines	>16	1 (2%)	0	ns
Metronidazole + clarithromycin		5 (8%)	3 (7%)	ns

Total		63/75 (84%)	46/66 (70%)	0.079
